# FGF-2b and h-PL Transform Duct and Non-Endocrine Human Pancreatic Cells into Endocrine Insulin Secreting Cells by Modulating Differentiating Genes

**DOI:** 10.3390/ijms18112234

**Published:** 2017-10-25

**Authors:** Giulia Donadel, Donatella Pastore, David Della-Morte, Barbara Capuani, Marco F. Lombardo, Francesca Pacifici, Marco Bugliani, Fabio A. Grieco, Piero Marchetti, Davide Lauro

**Affiliations:** 1 Department of Systems Medicine, University of Rome Tor Vergata, 00133 Rome, Italy; donatella.pastore@uniroma2.it (D.P.); david.dellamorte@uniroma2.it (D.D.-M.); barbara.capuani@uniroma2.it (B.C.); pacifici.francesca@gmail.com (F.P.); d.lauro@med.uniroma2.it (D.L.); 2 Department of Human Sciences and Quality of Life Promotion, San Raffaele Roma Open University, 00166 Rome, Italy; 3 Agenzia regionale per la protezione ambientale (ARPA) Lazio, Sezione di Roma, 00173 Rome, Italy; marcofelice.lombardo@poste.it; 4 Endocrinology and Metabolism of Transplantation, Azienda Ospedaliero-Universitaria (A.O.U.) Pisana, 56126 Pisa, Italy; m.bugliani@ao-pisa.toscana.it (M.B.); piero.marchetti@med.unipi.it (P.M.); 5 Department of Medicine, Surgery and Neuroscience, University of Siena, 53100 Siena, Italy; fagrieco@ulb.ac.be

**Keywords:** pancreatic β cells, cellular differentiation, insulin release, regenerative medicine, diabetes mellitus

## Abstract

**Background:** Diabetes mellitus (DM) is a multifactorial disease orphan of a cure. Regenerative medicine has been proposed as novel strategy for DM therapy. Human fibroblast growth factor (FGF)-2b controls β-cell clusters via autocrine action, and human placental lactogen (hPL)-A increases functional β-cells. We hypothesized whether FGF-2b/hPL-A treatment induces β-cell differentiation from ductal/non-endocrine precursor(s) by modulating specific genes expression. **Methods:** Human pancreatic ductal-cells (PANC-1) and non-endocrine pancreatic cells were treated with FGF-2b plus hPL-A at 500 ng/mL. Cytofluorimetry and Immunofluorescence have been performed to detect expression of endocrine, ductal and acinar markers. Bromodeoxyuridine incorporation and annexin-V quantified cells proliferation and apoptosis. Insulin secretion was assessed by RIA kit, and electron microscopy analyzed islet-like clusters. **Results:** Increase in PANC-1 duct cells de-differentiation into islet-like aggregates was observed after FGF-2b/hPL-A treatment showing ultrastructure typical of islets-aggregates. These clusters, after stimulation with FGF-2b/hPL-A, had significant (*p* < 0.05) increase in insulin, C-peptide, pancreatic and duodenal homeobox 1 (PDX-1), Nkx2.2, Nkx6.1, somatostatin, glucagon, and glucose transporter 2 (Glut-2), compared with control cells. Markers of PANC-1 (Cytokeratin-19, MUC-1, CA19-9) were decreased (*p* < 0.05). These aggregates after treatment with FGF-2b/hPL-A significantly reduced levels of apoptosis. **Conclusions:** FGF-2b and hPL-A are promising candidates for regenerative therapy in DM by inducing de-differentiation of stem cells modulating pivotal endocrine genes.

## 1. Introduction

Diabetes mellitus (DM) is a chronic multifactorial disease, characterized by a decrease in glucose-stimulated insulin secretion caused by pancreatic β cells dysfunction [1,2,3,4]. Type 1 diabetes mellitus (T1D) is induced by an autoimmune or idiopathic destruction of β cells [5,6,7,8], while type 2 diabetes mellitus (T2D) is mainly attributable to reduced β cell compensation in a setting of insulin resistance [5,6,9,10,11,12]. DM affects 371 million people worldwide [13,14]; therefore, novel preventive and therapeutic strategies are imperative [15].

In recent decades, stem cell therapy has been raised as a novel strategy for treatment of DM, with the final goal of reprogramming non-endocrine tissues into insulin-producing cells, achieving exciting results [16,17,18,19,20]. Although, in adults, the presence of stem/progenitor cells is well-documented in several tissues, e.g., bone marrow, skin, intestine and testis, the presence of stem/progenitor cells in human pancreas remains elusive [15,21,22,23,24]. Of note, it has been shown that β cells can be generated not only during fetal development, but also in adults life [25]. This phenomenon can occur in physiological conditions such as pregnancy, where increase of β cell mass is achieved by an enhancement of β cell numbers in apparently new small islets, rather than duplication of existing islets [26,27,28]. A similar process has been reported in pathological states, such as obesity, in which an increment of functional β cell mass results directly from an improvement in islet cell clusters associated to pancreatic ducts [9,29,30].

It is now well-established that new pancreatic islets can derive from different progenitor cells using various stimuli [12,31,32,33]. Promising strategies include differentiation of several type of cells, including embryonic or adult stem cells, mesenchymal cells, islet-derived progenitor cells, and trans-differentiation of differentiated adult cells [28,34,35,36,37]. The difficulty in identifying β cell progenitor(s) is manly based on the presence of different precursors by which they can originate [38,39]. β cell precursor(s) have been suggested to be present into ductal lining, or, alternatively, adult ductal pancreatic epithelial cells can differentiate into any pancreatic cell type [40,41]. This phenomenon of differentiation can occur from a cell de-differentiation process to a less mature phenotype [42,43]. Ducts, acini, and islets can then originate from these less differentiated cells, as already shown in a model of pancreas regeneration [40,44,45]. Based on this evidence, cells of ductal origin have been repeatedly used for assaying the ability of a number of molecules, mainly growth factors and hormones, to induce and expand in vitro insulin producing cells [46,47,48,49].

The mechanisms underlay islet-neogenesis are complexes and involve signaling and cell-cycle regulators, pancreatic islet specific transcription factors, and growth factors [50,51,52]. In particular, human fibroblast growth factor (FGF)-2b signaling has been implicated in patterning proliferation and cell differentiation in many organs including the developing pancreas [53,54]. Attenuation of FGF receptor 1c signaling in pancreas leads to reduced β cells number, impaired glucose sensing and perturbed proinsulin processing [55]. Furthermore, FGF-2b modulates the development and differentiation of endocrine pancreas through cell aggregation and cluster formation via autocrine/paracrine actions [56]. Placental lactogen (PL) hormone is a member of growth hormone and prolactin family. It plays a central role in maternal food intake and insulin production along with prolactin and placental growth hormone [57]. PL is produced by the placenta during pregnancy, and is considered the main factor responsible for mass increase of pancreatic islets and for improvement of their function during pregnancy [27,58]. Mouse PL expression in pancreatic β cells results in hyperplasia and hypertrophy of the organ, due to β cell increase in survival and proliferation [59].

Therefore, based on previous findings, the scope of this work was to verify whether treatment with: (i) FGF-2b; (ii) human placental lactogen (hPL)-A; or (iii) FGF-2b plus hPL-A (FGF-2b/hPL-A) can modulate the differentiation of adult non-endocrine/ductal pancreatic cells toward a β cell phenotype. To reach these goals, human pancreatic ductal-cells (PANC-1) cell line and non-endocrine cells isolated from human pancreas were used.

## 2. Results

### 2.1. Treatment with Human Fibroblast Growth Factor (FGF)-2b and Human Placental Lactogen (hPL)-A Promote Differentiation of Human Pancreatic Ductal-Cells (PANC-1) into Islet-Like Cell Aggregates

**Figure 1:**
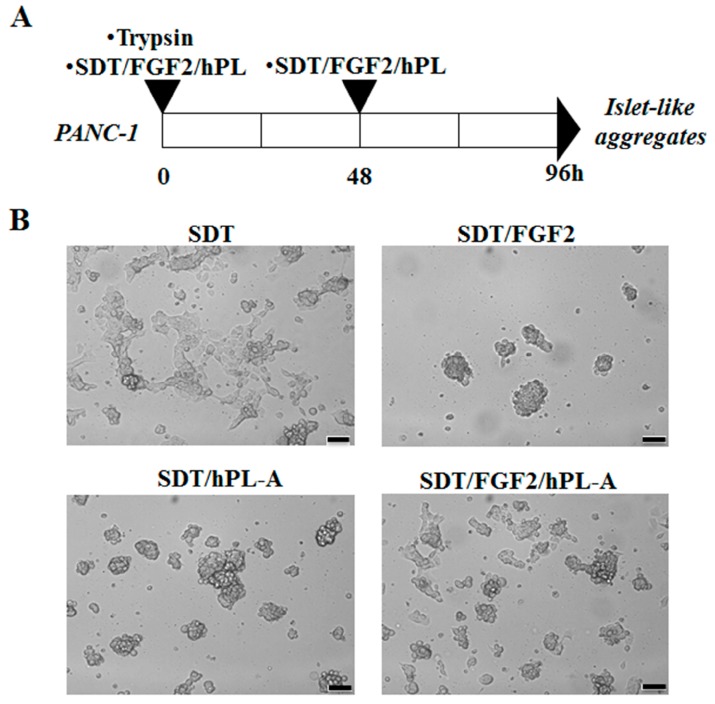
Induction of human pancreatic ductal-cells (PANC-1) differentiation towards islet-like aggregates. PANC-1 cells were treated with 50% trypsin for 30 s and incubated with serum-free medium supplemented with 0.1% bovine serum albumine (BSA) plus 1.1 mg/L transferrin (SDT). SDT medium was then supplemented with 500 ng/mL human fibroblast growth factor (FGF)-2b or 500 ng/mL human placental lactogen (hPL)-A, or with both hormones (FGF-2b plus hPL-A). Treatments were refreshed after 48 h, according to the scheme shown in (**A**). After 96 h, PANC-1 cells differentiated into islet-like aggregates (**B**). Scale bar = 100 μm.

With the aim of testing whether FGF-2b and hPL-A promote differentiation in human PANC-1 cells into islet-like aggregates, PANC-1 cells were treated with trypsin and then incubated in a serum-free medium containing transferrin (SDT), plus FGF-2b and/or hPL-A (Figure 1A). Generation of islet-like aggregates was achieved after stimulation of PANC-1 cells for 96 h with FGF-2b and hPL-A, alone and in combination, both at the same concentration of 500 ng/mL (Figure 1A,B) [60]. An improvement in the quality of islet-like aggregates was also noted when FGF-2b and/or hPL-A stimuli were refreshed after 48 h (data not shown). Differently, the treatment with SDT alone generated smaller and irregular-shaped scattered cells (Figure 1B), further suggesting that the effect of the cell transformation was associated to FGF-2b and/or hPL-A treatment.

### 2.2. Markers of Endocrine β Cells Are Modulated in Transformed Islet-Like Cell Aggregates after FGF-2b/hPL-A Treatment

**Figure 2:**
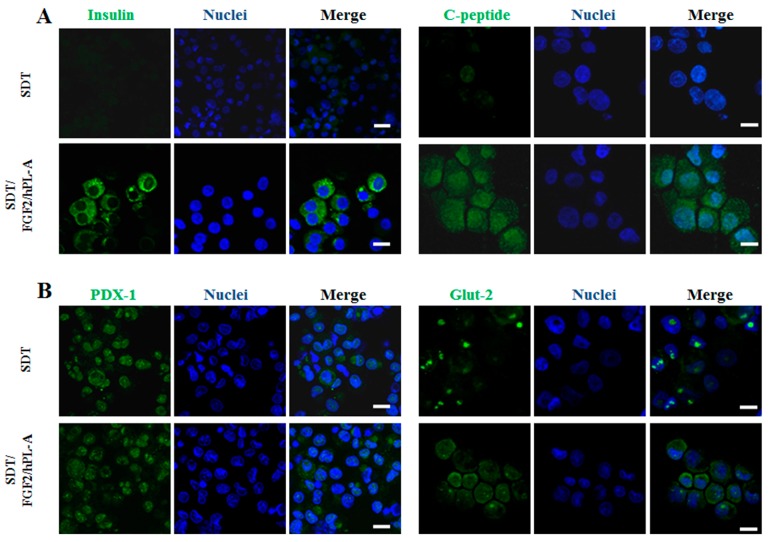
Confocal microscopy analysis of islet-like aggregates. Islet-like aggregates obtained after 96 h of treatment of PANC-1 cells with FGF-2b plus hPL-A were disaggregated to form single cell suspensions. Cells were then centrifuged with a cytospin on polilysine-coated slides and immediately fixed by paraformaldehyde 4%. After permeabilization, cells were green stained for: insulin and C-peptide (**A**); and glucose transporter 2 (Glut-2) and pancreatic and duodenal homeobox 1 (PDX-1) (**B**), while nuclei were blue-stained by Hoechst. Confocal microscopy analysis showed that treatment with FGF-2b plus hPL-A induced an increase of insulin and C-peptide staining in the cytosol, and a slight increase of PDX-1 staining in the nucleus and Glut-2 in plasma membrane. Scale bar = 20 μm.

Once PANC-1 duct cells differentiated into islet-like cell aggregates, their transformation in endocrine β cells was analyzed. Therefore, islet-like cell aggregates were collected and disaggregated into single cells, and then were analyzed with confocal microscopy to detect the expression of insulin, C-peptide, glucose transporter 2 (Glut-2), and pancreatic and duodenal homeobox 1 (PDX-1), classic endocrine/β cell markers [61,62,63]. After treatments with FGF-2b or hPL-A single treatment, a moderate, but not significant, increment of these markers was found (data not shown) compared to cells treated with SDT, used as control for this experiment. Conversely, following stimulation with the combination of FGF-2b plus hPL-A, a strong increment of insulin and C-peptide content in the cytoplasm and Glut-2 content in the plasma membrane was evident in comparison to SDT (Figure 2A,B). However, a slight enhancement of signal in PDX-1 staining in the nucleus and in the plasma membrane of islet-like cell aggregates compared to SDT, was clearly reported after hormonal combined treatment (Figure 2B).

**Figure 3:**
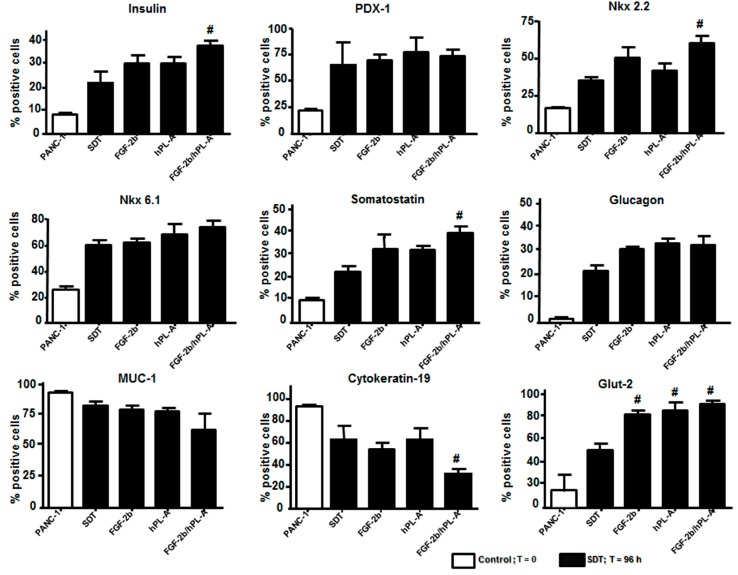
Cytofluorimetric analysis of islet-like aggregates. PANC-1 cells were treated with 50% trypsin for 30 s and incubated with serum-free medium supplemented with 0.1% BSA (0.1%) plus 1.1 mg/L transferrin (SDT). Analysis was expressed as percentage of positive cells in association with the specific markers. SDT medium was then supplemented with 500 ng/mL FGF-2b or 500 ng/mL hPL-A, or both hormones (FGF-2b plus hPL-A). After 96 h, islet-like aggregates were disaggregated to form single cell suspensions. Then, cells were fixed, permeabilized and stained for insulin, PDX-1, Nkx2.2, Nkx6.1, somatostatin, glucagon, MUC-1, Cytokeratin-19, and Glut-2, and immediately acquired on a BD FACSCalibur (at least 5 × 10^4^ event). # *p <* 0.05 vs. SDT; *n* = 4 (four separate experiments). Control: untreated PANC-1 cells.

To corroborate these results, FACS analysis was employed and expression of β cell/endocrine and duct markers was measured by considering the percentage of positive cells reactive to the specific markers evaluated. Treatment with FGF-2b, hPL-A, and FGF-2b/hPL-A combination at 500 ng/mL for 96 h in islet-like cell aggregates showed significantly increased levels (*p* < 0.05) of Glut-2 vs. SDT, while insulin, Nkx 2.2 and Somatostatin showed significantly (*p <* 0.05) increased levels vs. SDT only when treated with FGF-2b/hPL-A combination. Conversely, the ductal marker Cytokeratin-19 decreases significantly (*p <* 0.05) vs. SDT, while PDX-1, Nkx6.1, Glucagon and MUC-1 did not show a significant change vs. SDT. PANC-1 were untreated cells used as control (Figure 3).

**Figure 4:**
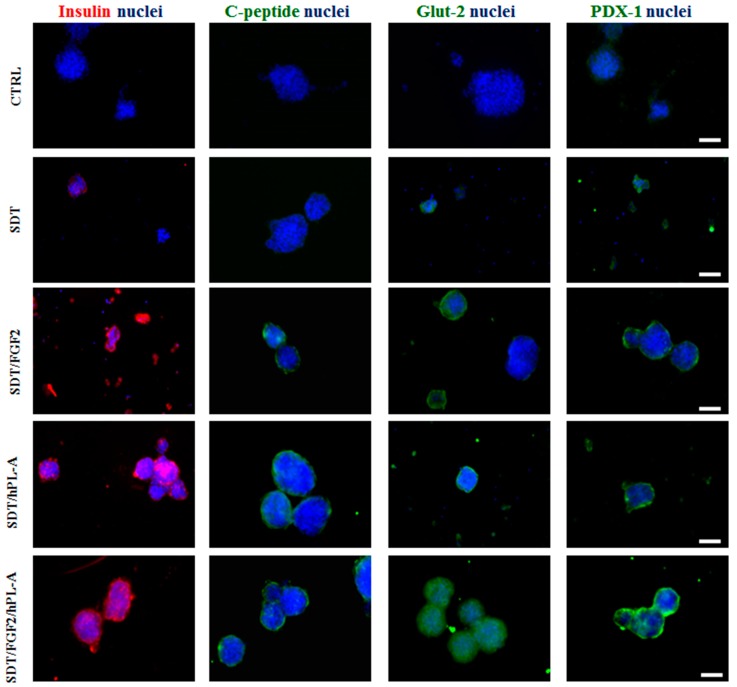
Immunofluorescence analysis of islet-like aggregates obtained from non-endocrine pancreatic cells isolated from healthy donors. Non-endocrine pancreatic cells obtained from healthy donors after the procedure for islet isolation were incubated with serum-free medium supplemented with 0.1% BSA plus 1.1 mg/L transferrin (SDT). SDT medium was then supplemented with 500 ng/mL FGF-2b or 500 ng/mL hPL-A, or both hormones (FGF-2b/hPL-A). After 48 h, stimuli were renewed and, following 96 h, cell aggregates were fixed and stained for the expression of insulin (**red**), C-peptide, Glut-2, and PDX-1 (**green**). Nuclei were blue-stained by Hoechst. Scale bar = 50 μm. CTRL: control, untreated non-endocrine pancreatic tissue from healthy donors.

We also validated these results in non-endocrine tissue obtained from pancreas of six Caucasian healthy donors (mean age, 53 ± 2.1 years; gender, four Male and two Female) recruited from Endocrinology and Metabolism of Transplantation, A.O.U. Pisana, Pisa, Italy. The paucity and preciousness of donor tissue give us the possibility to obtain material only for immunofluorescences and FACS analysis. Cell suspension was incubated with FGF-2b and/or hPL-A for 96 h. Immunofluorescence analysis in aggregate cluster of human cells showed the increased expression of insulin and Glut-2, and lower expression of C-peptide and PDX-1 (Figure 4). The double treatment with FGF-2b/hPL-A further increased the cells aggregation suggesting similar results in the human model would be obtained with PANC-1 cell cultures.

**Figure 5:**
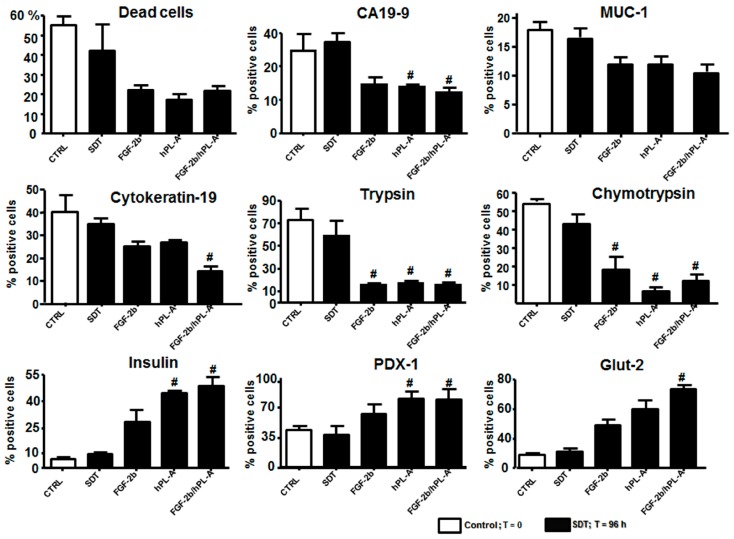
Cytofluorimetric analysis of islet-like aggregates obtained from non-endocrine pancreatic cells isolated from healthy donors. Non-endocrine pancreatic cells obtained from healthy donors after the procedure for islet isolation were immediately analyzed and then incubated with SDT alone, and SDT supplemented with 500 ng/mL FGF-2b, or 500 ng/mL hPL-A, or both hormones (FGF-2b plus hPL-A). Analysis was expressed as percentage of positive cells in association with the specific markers. After 48 h, stimuli were renewed and, following 96 h, aggregates were harvested and disaggregated into single cell suspensions. We performed the same panel of analyses before (*t* = 0) and after (*t* = 96 h) treatments: FACS for cell viability (propidium iodide) and for the expression of ductal markers Cytokeratin-19 and CA19.9, acinar markers chymotrypsin and trypsin, β cell markers insulin, PDX-1 and Glut-2, and adenocarcinoma marker MUC-1. # *p <* 0.05 vs. SDT; *n* = 4 (four separate experiments). CTRL: control, untreated non-endocrine pancreatic tissue from healthy donors.

Then, non-endocrine pancreatic cells tissue was disaggregated to obtain a single cell suspension to conduct FACS analysis. Cytofluorimetric analysis showed that FGF-2b plus hPL-A and/or single hormone treatments have a significant trend in reducing the rate of cellular death compared to SDT. After single and combined treatment, MUC-1 did not show any significant change in its level compared to SDT, while ductal/adenocarcinoma (CA19-9 and CK-19) and acinar (trypsin and chymotrypsin) markers were significantly (*p <* 0.05) reduced after hormonal treatment compared to SDT. Conversely, a significant (*p <* 0.05) increased expression of β cell markers (insulin, PDX-1, and Glut-2) was evident after FGF-2b plus hPL-A treatment compared to SDT (Figure 5). These results also established that human differentiated cells are biologically active after hormonal treatments, and therefore potentially useful in the models of regenerative medicine.

### 2.3. Treatment with FGF-2b and hPL-A Inhibits Cellular Growth and Reduces Apoptosis in Islet-Like Aggregates

**Figure 6:**
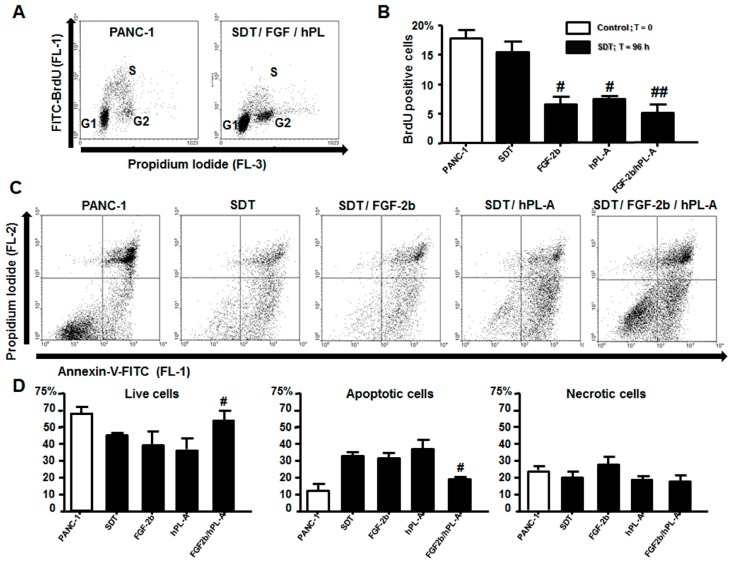
Islet-like aggregates were analyzed for bromodeoxyuridine (BrdU) incorporation and apoptosis. PANC-1 cells were treated with 50% trypsin for 30 s. and incubated with serum-free medium supplemented with 0.1% BSA plus 1.1 mg/L transferrin (SDT). SDT medium was then supplemented with 500 ng/mL FGF-2b or 500 ng/mL hPL-A, or both hormones (FGF-2b plus hPL-A). After 96 h of treatment, islet-like aggregates were incubated for additional 20 min. with BrdU. Then, aggregates were harvested, disaggregated to form a single cell suspension and immediately analyzed using FACS. (**A**) Typical dot plots are shown: note the decrement in the BrdU-positive (S-phase) cell number induced with FGF-2b plus hPL-A treatment. (**B**) The percentages of S-phase cells after 96 h of incubation are shown. # *p <* 0.05 and ## *p <* 0.01 vs. SDT; *n* = 4 (four separate experiments). For the analysis of apoptosis, the single cell suspensions were incubated with annexin-V-FITC and propidium iodide, and immediately analyzed with FACS. In the dot plots (**C**), we can identify live cells (low left quadrant), apoptotic cells (low right quadrant), and necrotic cells (upper quadrants). (**D**) The percentages of live, apoptotic and necrotic cells are shown. # *p <* 0.05 vs. SDT; *n* = 4 (four separate experiments).

Successively, we tested whether treatment with FGF-2b and/or hPL-A had an impact on the islet-like aggregates, obtained from PANC-1, in term of cellular growth, apoptosis and necrosis. Islet-like aggregates after treatment for 48 h and 96 h with SDT plus FGF-2b and/or hPL-A were analyzed by FACS following double staining with bromodeoxyuridine (BrdU)-FITC/Propidium iodide, which enables the identification of cells in G1-, G2-, and S-phases (Figure 6A,B). We tested apoptosis using the Annexin-V/Propidium iodide staining in order to evaluate early apoptosis, necrosis and living cells on the same samples treated for 96 h with SDT, FGF-2b, hPL-A and FGF-2b plus hPL-A. FGF-2b plus hPL-A treatment was able to significantly increase (*p <* 0.05) the number of live cells (Figure 6C,D), and to significantly decrease (*p <* 0.05) apoptotic cells (Figure 6C,D), compared to cells incubated with SDT alone. No significant differences among treatments were found regarding number of necrotic cells (Figure 6C,D).

### 2.4. FGF-2b Plus hPL-A Treatment Changes Ultrastructure and Biological Activity of PANC-1 Cells

**Figure 7:**
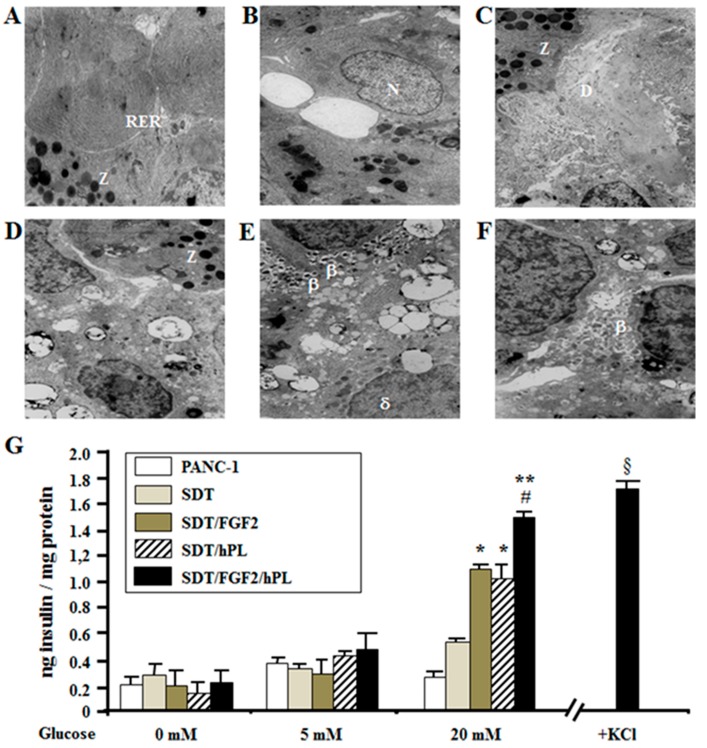
Ultrastructure of islet-like aggregates and insulin secretion assay. PANC-1 cells (70% of confluence) were treated with 50% trypsin for 30 s. and incubated with serum-free medium supplemented with 0.1% BSA plus 1.1 mg/L transferrin (SDT). SDT medium was then supplemented with 500 ng/mL FGF-2b or 500 ng/mL hPL-A, or both hormones (FGF-2b plus hPL-A). After 96 h, aggregates were harvested, fixed and analyzed by electron microscopy. SDT aggregates were composed by acinar cells (Z, zymogen granules; RER, endoplasmic reticulum (**A**). The ultrastructure of cell aggregates obtained with FGF-2b and hPL-A treatment, respectively (N, nucleus; Z, zymogen granules; D, ductal cell) (**B**,**C**) . Cell aggregates obtained with FGF-2b plus hPL-A treatment showed the presence of acinar cells (Z, zymogen granules (**D**)) in the border; and a core composed by some: δ cells (δ (**E**)); and β cells with: mature (β (**E**)); and immature (β (**F**)) granules. Insulin secretion assay is shown (**G**): after 96 h of treatment, aggregates were washed and incubated in Krebs/HEPES buffer supplemented with 0.1% BSA for 3 h. Then, glucose 5 mM or 20 mM (or KCl 300 μM) was added for 1 h to stimulate insulin secretion. Glucose-induced insulin secretion was analyzed with insulin-RIA kit and normalized for the total protein content. * *p <* 0.05 and **p < 0.01 vs. both PANC-1 and SDT; # *p <* 0.05 vs. both FGF-2b and hPL-A; § *p <* 0.05 vs. FGF2b/hPL-A; *n* = 4 (four separate experiments).

To further confirm previous results, electron microscopy analysis was performed on islet-like aggregates after 96 h of incubation with different treatments, with the aim to analyze cellular ultrastructure transformation after these stimuli. As shown in Figure 7A, SDT induced formation of acinar-like cells with the presence of zymogen granules (Z) and an expanded endoplasmic reticulum (RER), but no evidence of endocrine cells. FGF-2b treatment leaded to the formation of mesenchymal cells and duct-like structures (Figure 7B), while hPL-A treatment induced the formation of acinar-like cells mainly in the border of aggregates, and mesenchymal and ductal cells in the core (Figure 7C). After treatment with FGF-2b plus hPL-A, islet-like aggregates showed a complex structure with the border composed of acinar-like cells (Figure 7D) and the core of islet-like cells, mainly β-like cells. Almost 80% of β-like cells contained mature insulin granules (β; Figure 7E) and some δ-like cells (δ; Figure 7E), and some α-like cells were also found (data not shown). While the remaining β-like cells contained immature granules (β; Figure 7F). Cell specific granules are considered cellular specific markers to identify β-like cells when identified by using the electron dense core [64], as we used in the present study.

To analyze whether β-like cells present in the aggregates were active and therefore able to secrete insulin, they were stimulated with glucose (5 mM/1 h or 20 mM/1 h). Glucose 20 mM/1 h increased insulin secretion in these β-like cells after FGF-2b plus hPL-A treatment compared with SDT (*p <* 0.01) and with FGF2b or hPL-A single treatment (*p <* 0.05). However, cells treated with FGF2b or hPL-A showed higher levels of insulin secretion compared with SDT incubated cells (*p <* 0.05) (Figure 7G), suggesting an effect of these factors in β-cells secretion.

To better characterize the transformation of PANC-1 in β-like cells, we evaluated by immunofluorescence analysis the PDX-1 expression and localization after stimulation with glucose (20 mM/1 h) and FGF-2b plus hPL-A treatment (Figure S1). We found that, after glucose stimulation in treated PANC-1 cells, PDX-1 increased its translocation from cytoplasm to nucleus compared to no-glucose stimulated cells, supporting the induction of transformation of these cells into endocrine insulin productive cells mediated by hormonal stimulation.

## 3. Discussion

This study reports that treatment of human PANC-1 ductal cells with hPL-A and FGF-2b induces a deep differentiation of these cells resulting in generation of islet-like aggregates. These aggregates express typical islet markers, and acquire the ability to secrete insulin in response to glucose, supporting the differentiation of these cells into endocrine organ. Although the amount of secreted insulin is lower compared to physiological pancreatic β cells, the finding presented in this study may open a novel therapeutic strategy in the field of regenerative medicine.

Previous studies already reported the potential of PANC-1 to differentiate into islets of Langerhans. PANC-1 duct cells exposed to a mild treatment with trypsin and incubated in serum-free medium (SFM) in the presence of insulin, selenium and transferrin underwent the formation of hormone expressing islet-like aggregates [56]. In agreement with our data, SDT treatment alone was able to activate the expression of β cells/endocrine markers and to reduce ductal markers. Interestingly, here we showed that treatment with combined hormonal treatment induced stronger expression of β cells/endocrine markers compared to SDT, FGF-2b or hPL-A single treated cells. A similar behavior was observed in the reduction of ductal markers. Taken together, our data suggest that a multi stimuli approach could be useful for a complete differentiation from ductal progenitors to β cells. In fact, in our study, PANC-1 cells cultured in SDT without insulin and selenium were not able to fully differentiate into islet-like aggregates. Conversely, after treatment with FGF-2b, they differentiated into islet-like aggregates. The important role of FGF-2b in modulating β cell physiology has been further established by demonstrating that its lower levels lead to reduced β cell mass, impaired glucose sensing and perturbed proinsulin processing [55]. Moreover, the role in regenerative medicine of FGF-2b has been demonstrated thorough the differentiation of undifferentiated precursors in pancreas and heart: (i) in PDX-1^+^ pancreatic progenitor cells, through the activation of MAPK signaling pathway [54]; and (ii) in functional cardiomyocytes, with any effect on the precursors number and without inhibiting mitogenesis [65]. These results are agreement with data from our study since we did not find any increment of cell proliferation after treatment with FGF-2b, as well as with hPL-A or with FGF-2b/hPL-A combination.

PL is the putative major hormone implicated in enhanced islet mass and function, which occurs during pregnancy [27,58]. Recently, we showed a secretagogue activity of this hormone on cultured human islets [60]. Furthermore, it has also been reported that PL can induce β cell proliferation and, likely, neogenesis through the production of serotonin [66,67], which in turn might induce β cell neogenesis in ductal/non endocrine cells. To our knowledge, even with this important function, the role of hPL-A, as possible factor to induce differentiation of different cell lines into islet-like cell aggregates, was never tested before.

In mice, two major mechanisms account for pancreatic islet regeneration, namely β-cell replication and differentiation [68]. In contrast, in humans, there is almost no β-cell replication and the increased β-cell mass appears to be regulated through new islet formation derived from exocrine/ductal precursor cells [10,51,69,70]. PANC-1 cell line is a human pancreatic carcinoma, epithelial-like cell line, which presents ductal cell characteristics with higher amount of cells positive to the duct cell markers CK19 (~70%), MUC-1 and c-Kit, and with the presence of undifferentiated cell populations positive for alkaline phosphatase and α-fetoprotein [47]. Thus, PANC-1 is a heterogeneous, relatively undifferentiated cell population, and it is possible to hypothesize the existence of similar precursor/stem cells in human pancreas. FGF-2b and hPL-A may promote islet regeneration in vivo similarly to GLP-1 and IGF-1 [48,58]. However, GLP-1 promotes the differentiation of ductal cells into pancreatic β-like cells only in cells already expressing PDX-1 [48], while, in our experimental conditions, FGF-2b and/or h-PLA can promote the expression of PDX-1 in PANC-1 and in non-endocrine human cell lines.

PDX-1 is the foremost regulator of pancreatic development as well as of β cell differentiation and maintenance of mature β cell phenotype, both in humans and in rodents [71,72]. Furthermore, PDX-1 can stimulate insulin synthesis and secretion in various non-β cell types [73]. PDX-1 can activate the expression of the homeodomain transcription factors Nkx2.2 and Nkx6.1 that are typically expressed in the pathway regulating pancreatic β cell formation and, importantly, are maintained in mature β cells [74]. Therefore, their activation could trigger the differentiation of PANC-1 and non-endocrine pancreatic cells towards an endocrine β cell like phenotype. In our experimental conditions, we observed an slight enhancement of PDX-1 expression measured by immunofluorescence analysis in PANC-1, while a significantly enhancement (*p* < 0.05) in non-endocrine pancreatic cells isolated from healthy donors by immunofluorescence and FACS analysis. This result, along with increase of insulin, and somatostatin in response to FGF-2b plus hPL-A in PANC-1 cells, suggests that these hormones can commit the differentiation of duct/non endocrine cells toward an endocrine phenotype generating islet-like aggregates. Moreover, after glucose stimulation, it was observed an increase of PDX-1 nuclear translocation in treated PANC-1 cells, suggesting the switch into β cell phenotype of these cells.

In the present study, after incubation with FGF-2b and/or hPL-A, cell replication rate decreased in all treated ductal cells, with a reduced number of cells in S-phase. It is well known that differentiation of mammalian cells is associated with replicative quiescence, and that the more differentiated cells do not proliferate [75]. Therefore, the decreased levels of S-phase in treated cells may be interpreted as an arrest in cell replication associated with higher apoptotic processes after treatment with single hormones (SDT, FGF-2b or hPL-A). Differently, treatment with FGF-2b plus hPL-A significantly reduced cell apoptosis suggesting the induction of cell differentiation process.

Cells treated with FGF-2b plus hPL-A were able to synthesize and secrete insulin in response to glucose, as showed with C-peptide and insulin antibody cells staining and as measured in cells medium using insulin radioimmunoassay, further suggesting the powerful effect of combined hormonal treatment vs. the single hormonal treatment. Then, electronic microscope analysis showed aggregates obtained from cells treated with combination factors shared many features similar with human islets. We observed that the core of islet-like aggregates were mainly composed by cells displaying an ultrastructure similar to pancreatic β cells, with the presence of insulin granules (markers of differentiation) at different stages of maturation. Furthermore, these islet-like aggregates have a significant decrease of CK-19 expression, which is a typical adenocarcinoma/ductal marker, and an increment of endocrine markers expression. Even if the concept that non-endocrine cells can be induced to produce and secrete insulin in response to glucose is not novel [76], in the present study, we showed hormonal treated ductal PANC-1 and human non-endocrine cells, obtained from healthy donors, lose their typical phenotype (monolayer, CK19^+^/MUC-1^+^, trypsin^+^ and chymotrypsin^+^) and gradually express typical endocrine/β cell phenotype (islet-like cell clusters, insulin^+^/C-peptide^+^/nuclear PDX-1^+^/Glut-2^+^, glucagon^+^, somatostatin^+^).

In human ductal cells isolated from donors, we found a similar activation of β cell-markers. However, these results are preliminary due to difficulties in recruit the biological materials from donors and to the technical difficulties in long term culturing of these cells. Further studies are imperatives to improve and confirm our results in humans.

Present results are in agreement with several in vitro and in vivo studies suggesting epithelial ductal layer could be a promising source of insulin secreting cells [69,77,78,79,80]. In particular, the existence of insulin^+^/CK-19^+^ cells has been previously shown in the ducts of transplanted pancreas, specifically in T1D patients with recurrent autoimmunity [81]. These cells also stained for PDX-1, and, interestingly, the patients with the most severe β cells loss showed the highest number of insulin-positive ductal cells in the transplanted pancreas. This indicated how diabetes mellitus and chronic autoimmunity/inflammation might trigger pancreas remodeling by activating ductal cell proliferation and differentiation. Moreover, it has been shown that mouse and human acinar cells can generate duct cells through activation of acino-ductal transdifferentiation [80]. It could also be possible, in diabetes, that acinoductal transdifferentiation may occur and contribute to the process of regeneration of a functional pancreatic β cell mass [82]. We also confirmed that human acinar and ductal tissues can transdifferentiate into insulin-positive cells in vitro, as already reported [81,83].

Noteworthy, electron microscopy analysis of islet-like aggregates from PANC-1 showed presence of mesenchymal cells. In particular, when the differentiation process was not completed and cell aggregates were not tight and organized, were found few isolated β cells and the presence of large numbers of mesenchymal cells (not shown). This observation could suggest the occurrence of epithelial to mesenchymal transition (EMT). Islet-derived stem/precursor cells (IPCs) have been reported to be a subpopulation of the mesenchymal-like stem cells within human islets with lack expression of ductal markers CA19-9 and CK-19 suggesting a non-ductal origin [19,84].

## 4. Materials and Methods

### 4.1. Cell Culture and Differentiation Protocol

The plasmid phPL4828 (human placental lactogen), coding for the recombinant hPL (isoform A) protein, was kindly provided by Genentech (San Francisco, CA, USA), and purified in our laboratory as previously described [85]. FGF2 (FGF-2b), transferrin and all powders were purchased by Sigma-Aldrich (St. Louis, MO, USA). Cell culture and molecular biology products were purchased from Invitrogen (Paisley, UK). Human pancreatic ductal cells PANC-1 (European Collection of Cell Cultures—Wiltshire, UK) were cultured for 72–120 h in Dulbecco’s modified Eagle’s medium (DMEM) high glucose 25 mmol/L supplemented with 10% FBS, 1% penicillin/streptomycin and incubated in humidified 5% CO_2_ and 95% air at 37 °C. At ~70% confluence, medium was removed, cells were washed and then treated with 50% trypsin/phosphate buffered saline (PBS) for 1 min, in order to loosen but not to detach the cellular monolayer from their extracellular matrix. Then, cells were incubated with low-glucose serum-free DMEM supplemented with 0.1% bovine serum albumine (BSA) plus 1.1 mg/L transferrin (SDT), and with SDT supplemented with 500 ng/mL FGF-2b, or 500 ng/mL hPL-A, or both hormones (FGF-2b/hPL-A).

### 4.2. Isolation of Human Non-Endocrine Pancreatic Cells

Human non-endocrine pancreatic cells were obtained from 6 non-diabetic multi organs donors (aged, 53 ± 2.1 years; gender, 4 Male and 2 Female). Following the procedure of islet isolation [86], the remaining non-endocrine material was mechanically disaggregated in order to obtain single cell suspensions. These cells were then stimulated with the same protocol used for PANC-1 cells.

### 4.3. Immunofluorescence Analysis

Islet-like aggregates obtained from PANC-1 were treated with 0.05% trypsin/Ethylenediaminetetraacetic acid (EDTA) to obtain a single cell suspension. Cells were then centrifuged at 1000 rpm for 10 min on microscope slides, immediately fixed with 4% paraformaldehyde in PBS for 20 min at room temperature (RT) and then permeabilized with 0.25% Triton X-100 for 10 min at RT. Islet-like aggregate staining was performed with rabbit anti-human PDX-1 (Millipore, Billerica Headquarters, Billerica, MA, USA), Guinea pig anti-human insulin, rabbit anti-human C-peptide (Linco Research, St. Charles, MO, USA), rabbit anti-human Glut-2 (Millipore, Billerica Headquarters, Billerica, MA, USA). Islet-like aggregates obtained from human pancreatic non-endocrine cells, were fixed in 4% paraformaldehyde for 20 min, then cells were washed twice with PBS and permeabilized in ice-cold 50% methanol/50% acetone for 5 min and washed. Cells were then incubated with blocking solution (5% BSA in PBS) for 30 min followed by 1 h incubation at RT with the following antibodies: Guinea pig anti-human insulin, rabbit anti-human C-peptide (Linco Research, St. Charles, MO, USA), rabbit anti-human Glut-2, rabbit anti-human PDX-1 (Millipore, Billerica Headquarters, Billerica, MA, USA). After, cells were washed and then incubated with the appropriate fluorescent secondary antibodies: PE-conjugated anti-Guinea pig (red staining; Jackson Lab.) and Alexa Fluor 488-conjugated anti-rabbit (green staining; Invitrogen). Negative controls were performed omitting primary antibodies. Nuclei were stained with Hoechst 33342 (Invitrogen). Immunofluorescence analysis was performed by a microscope Nikon Eclipse TE-2000S equipped with a cool-snap camera. Microscope LSM 510-Meta Zeiss and a Laser Confocal Microscope Leica TCS SP5 were used for confocal microcopy analysis.

### 4.4. Cytofluorimetric Analysis

Islet-like aggregates were detached and single cells disaggregated by trypsin treatment. Next, cells were fixed by 2% paraformaldehyde for 10 min and recovered by centrifuge (1000 rpm for 5 min). After washing twice with PBS, cells were permeabilized with PBS containing 1% of Tween-20 for 10 min at RT, washed again twice with PBS, and incubated with blocking solution (2% BSA, 10% FBS in PBS) for 10 min. After, cells were incubated for 1 h at RT with the following antibodies: Guinea pig anti-human insulin, rabbit anti-human glucagon (Linco Research, St. Charles, MO, USA); rabbit anti-human PDX-1, rabbit anti-human cytokeratin19 (CK-19; Millipore, Billerica Headquarters, Billerica, MA, USA); rabbit anti-human Nkx2.2, rabbit anti-human Nkx6.1 (S. Cruz Biotech. Inc., Heidelberg, Germany); and mouse anti-human MUC-1, mouse anti-human somatostatin (Ab-Serotec, Oxford, UK). Then cells were washed three times with PBS and incubated with the appropriate fluorescent secondary antibodies: PE-conjugated anti-Guinea pig (Jackson Lab.), Alexa Fluor 488-conjugated anti-rabbit or anti-mouse (Invitrogen). Isotype identical antibodies served as control. Then, cells were promptly analyzed with a BD FACSCalibur equipped with the CellQuest software.

### 4.5. Cell Growth and Apoptosis

Cell growth rate and apoptosis were analyzed using the S-Absolute assay kit (Millipore, Billerica Headquarters, Billerica, MA, USA) and the APO-AF kit (Sigma-Aldrich, St. Louis, MO, USA), respectively, on a BD FACSCalibur equipped with the CellQuest software.

### 4.6. Insulin Secretion Assay

Islet-like cell aggregates were incubated in Krebs/HEPES buffer supplemented with 0.1% BSA for 3 h, after buffer was removed, centrifuged and the supernatants were stored at −80 °C. Then, Islet-like aggregates were incubated in Krebs/HEPES buffer supplemented with 0.1% BSA and 5 mM or 20 mM glucose for 1 h, later glucose-supplemented buffer was removed, centrifuged and the supernatants were stored at −80 °C. Next, Islet-like aggregates were lysed and their total protein content was assessed using Bradford method. Insulin content in Krebs/HEPES buffer was assayed employing an RIA kit (GE Healthcare, Milano, Italy) and each value was normalized for its own protein content.

### 4.7. Electron Microscopy Analysis

Aggregates were fixed in 2.5% glutaraldehyde in 0.1M phosphate buffer for 20 min. at RT, washed in 0.1 M phosphate buffer (pH 7.3), post-fixed in 0.1% osmium tetroxide in 0.1 M phosphate buffer (pH 7.3) and dehydrated in a graded series of ethanol. Aggregates were then transferred to propylene oxide and embedded in PolyBed 812. Ultrathin sections were cut with a diamond knife, stained with uranyl acetate and lead citrate, and observed under a Zeiss 902 transmission electron microscope.

### 4.8. Statistical Analysis

Results are expressed as means ± SD. Statistical analysis was performed with ANOVA followed by Newman–Keuls post hoc test, using the software Prism GraphPad 4. Values of *p <* 0.05 were considered significant.

### 4.9. Ethical Approval

The use of Human tissue was approved by the local Ethical Committee of Pisa, Italy. According to the Italian legislation, organ and tissue donation is regulated by art. 23 of the national law n. 91, issued on 1 April 1999. The Tuscany regional transplant organization (OTT, Organizzazione Toscana Trapianti: http://www.parlamento.it/parlam/leggi/99091l.htm) allows that organs not suitable for clinical transplantation are used for research purposes. Since tissue belong to brain dead donors, provided informed consent has been signed by the next-of-kin. Prof. Marchetti’s group has complete access to donated pancreases for the preparation and study of isolated islets on the basis of approval by their local ethics committee, renewed in 2013 (N. 3897).

## 5. Conclusions

In summary, our study reveals that the combined treatment with hPL-A plus FGF-2b are promising factors to induce differentiation of duct/non-endocrine cells towards an endocrine phenotype. Our results confirm that ductal cells may represent an encouraging source of insulin producing cells. Furthermore, it is possible to speculate that hPL-A plus FGF-2b could be a new therapeutic approach in the field of regenerative medicine with the aim to restore a functional pancreatic β cell mass in patients with diabetes mellitus.

## Supplementary Materials

Supplementary materials can be found at www.mdpi.com/1422-0067/18/11/2234/s1.
