# Supplementary material for: FGF-2b and h-PL Transform Duct and Non-Endocrine Human Pancreatic Cells into Endocrine Insulin Secreting Cells by Modulating Differentiating Genes
Source: Int J Mol Sci. 2017 Oct 25;18(11):2234. doi: 10.3390/ijms18112234 (PMC5713204; doi:10.3390/ijms18112234)
Supplement: Supplementary file 1 [file ijms-18-02234-s001.pdf]

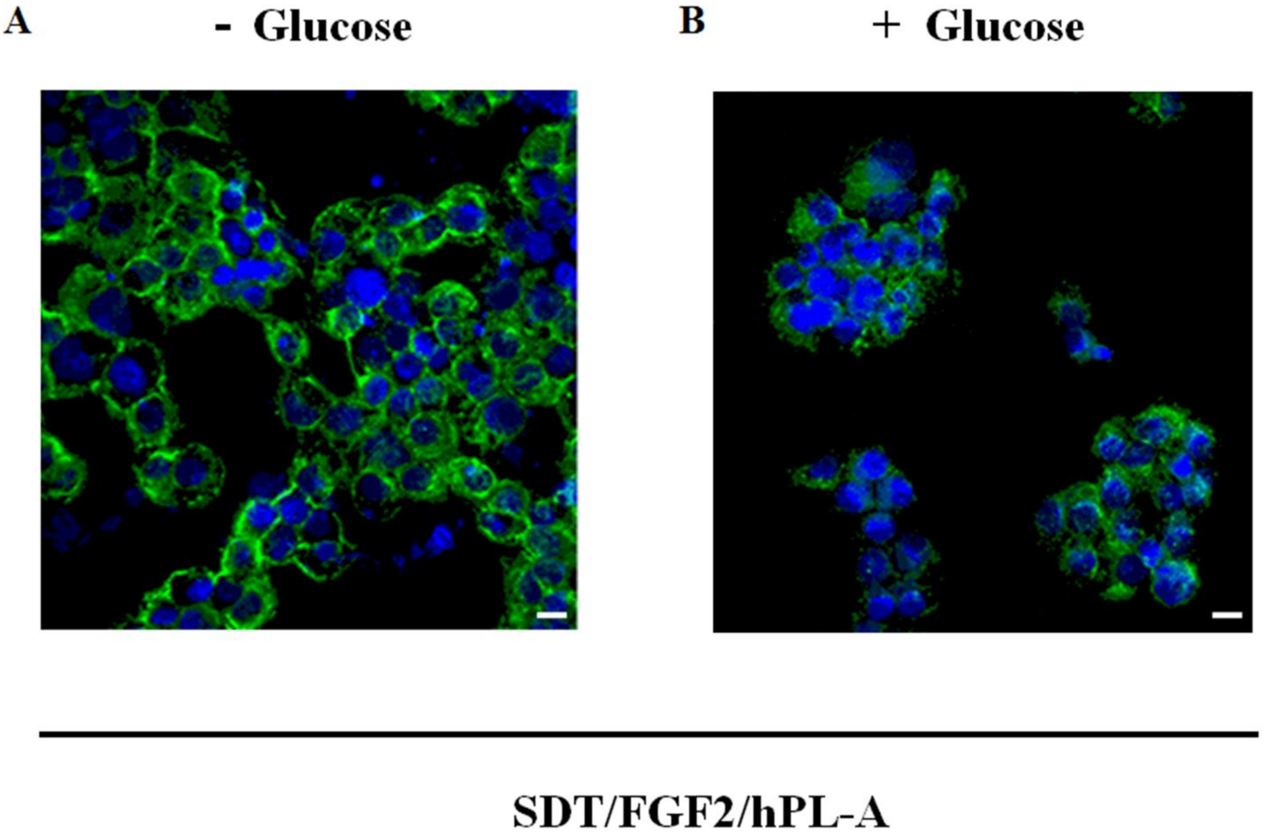

Supplementary Figure 1. *Confocal microscopy analysis of PDX-1 internalization.* Islet-like aggregates obtained after 96h of treatment of PANC-1 cells with FGF-2b plus hPL-A (A, B) and stimulated with glucose (20mM for 1 hours) (B), were disaggregated to form single cell suspensions. Cells were then centrifuged with a cytospin on polilysine-coated slides and immediately fixed by paraformaldehyde 4%. After permeabilization, cells were stained for PDX-1 (green) while nuclei were blue-stained by Hoechst (A, B). Confocal microscopy analysis showed that treatment with FGF-2b plus hPL-A induced an increase of PDX-1 staining in the nucleus, after stimulation with glucose (B) that induces PDX-1 internalization. Space bar = 20 μm.
